# Guidelines for Verification of Gastric Tube Location in Adult Hospitalised Patients: A Systematic Review

**DOI:** 10.1111/nicc.70466

**Published:** 2026-03-30

**Authors:** Cornelius Baving, Markus Grebe, Franziska Wefer, Lars Krüger, Sascha Köpke

**Affiliations:** ^1^ Faculty of Medicine and University Hospital Cologne, Institute of Nursing Science University of Cologne Cologne Germany; ^2^ Care Development, Care Directorate, Heart and Diabetes Center NRW Ruhr University Bochum Bad Oeynhausen Germany; ^3^ Project and Knowledge Management/Care Development Intensive Care, Care Directorate, Heart and Diabetes Center NRW Ruhr University Bochum Bad Oeynhausen Germany

**Keywords:** enteral nutrition, gastrointestinal, intubation

## Abstract

**Background:**

Verification of correct gastric tube placement is a major patient safety issue. Guidelines recommend different procedures.

**Aim:**

To evaluate international guidelines for verifying correct gastric tube placement in adult hospitalised patients, identifying recommended practices and methodological quality.

**Study Design:**

Systematic review with searches conducted in Medline, CINAHL and guideline repositories until January 2024. Reference lists of identified articles were also screened. Guidelines on adult gastric tube placement based on a systematic literature search and/or a formal consensus process were included. Data extraction and quality assessments, using the AGREE‐II tool, were conducted by two and four reviewers respectively.

**Results:**

Six guidelines from three countries were included. Radiographic confirmation is widely regarded as the gold standard for verifying gastric tube placement, especially when the position is uncertain, although bedside methods such as pH measurement, capnography and visual inspection are increasingly recommended. There is great variability regarding recommended bedside techniques, pH thresholds and routine monitoring practices. Marking the tube's exit site and routinely monitoring external length are universally recommended. Five guidelines recommend against the auscultation method either in general or at least with regard to its sole use. Methodological quality and evidence strength vary significantly across guidelines.

**Conclusions:**

While consensus exists on some aspects, variability in recommendations reflects inconsistent evidence bases. Most guidelines showed insufficient methodological quality. International standardisation and high‐quality primary research are necessary.

**Relevance to Clinical Practice:**

This review synthesizes current guidelines on gastric tube placement, exposing methodological weaknesses, recommendation inconsistencies and unreliability of auscultation, identifying a critical information gap in clinical practice.

## Impact Statements

- What is known about the topic
  ○ Correct gastric tube position is critical to prevent complications.
  ○ Radiographic confirmation is considered the gold standard, but bedside methods are commonly used.
  ○ Existing guideline reviews are outdated, and practices such as auscultation remain widespread despite limited reliability.
- What this paper adds
  ○ Provides an up‐to‐date synthesis of international guidelines on gastric tube placement verification.
  ○ Highlights significant methodological weaknesses and variation in recommendations across current guidelines.
  ○ Identifies auscultation as unreliable, thus challenging current practice.

## 1 Introduction

The use of nasogastric tubes is an established procedure in clinical practice for nutrition, medication administration and decompression of the gastrointestinal tract in hospitalized patients. In critical care, the insertion of a nasogastric tube is regularly performed by a nurse [1]. Verification of the correct tube placement is crucial to ensure that patients do not experience complications from the procedure [2].

## 2 Background

Gastric tubes are usually inserted by healthcare professionals, often at the bedside in various hospital departments such as emergency rooms, intensive care units or surgical wards. They are used for enteral feeding, administering medication or stomach decompression in patients who cannot safely eat or swallow, or who have undergone gastrointestinal surgery. The management of gastric tubes requires special care to ensure patient safety and comfort. Accurate placement, continuous monitoring, and the reduction of both physical and psychological stress are key tasks within interdisciplinary care and, especially, critical care nursing practice.

Despite its frequent use, the process of inserting a nasogastric tube carries significant risks. An integrative review of case reports detailing complications caused by nasogastric tubes emphasises that adverse events with this intervention are common and can cause serious health damage, leading to prolonged hospital stays or even death [3]. There are case reports of deaths and permanent physical damage associated with complications due to inadvertent placement of a feeding tube in the respiratory tract, the central nervous system [4], or the occurrence of a pneumothorax due to penetration, as well as aspiration [3, 5].

In a review of 9931 small‐bore nasogastric tube placements, 1.9% of the tubes were incorrectly placed in the respiratory tract, and at least five patients lost their lives due to complications from nasogastric tube insertion [6]. Similar studies showed comparable results [5]. Also, misplacement within the gastrointestinal tract increases the risk to patients. Placement or dislodgement of the nasogastric tube in the oesophagus raises the risk of aspiration [5]. Misplacement of the tube in the duodenum may promote the development of malabsorption or dumping syndrome [7].

Methods for verifying gastric tube placement include radiographic visualisation, pH measurement of aspirate, auscultation/palpation while air insufflation, capnometry/‐graphy using CO_2_ detectors, visual assessment of aspirate appearance, blind placement, ultrasound, ‘bubbling’, ECG guidance, external magnet guidance, ‘electromagnetic field detection’, illumination, bilirubin or enzymatic analysis of aspirate (e.g., pepsin/trypsin) and endoscopic confirmation under direct visual guidance [8].

Due to the numerous case reports of complications, researchers recommend that evidence‐based guidelines should be developed for the safe insertion and correct positioning of enteral tubes to improve patient safety and minimise complication rates [3, 9]. However, there is currently no specific guideline for verifying the position of gastric tubes in many health care systems, including the German one [10].

Furthermore, several years have passed since the last comprehensive review of the guidelines by Metheny et al. in 2019 [10]. Importantly, this work did not include a formal quality assessment of the guidelines, and it addressed both adult and paediatric populations. The review primarily highlighted the considerable variability in guideline recommendations that supported radiography as the most accurate method for confirming gastric tube placement, particularly in high‐risk patients. Non‐radiological methods such as pH testing were regarded as showing some promise, but lacking consensus and reliability, whereas other techniques such as auscultation and visual assessment were generally not recommended [10].

## 3 Objective of the Review

This systematic review aims to systematically assess specific recommendations from current international guidelines of national healthcare institutions and from professional healthcare societies for examining the position of nasogastric tubes in hospitals and the quality of these guidelines.

## 4 Design and Methods

A systematic review of current international guidelines for examining gastric tube position was conducted. The review was prospectively registered with PROSPERO [11]. The reporting follows The Preferred Reporting Items for Systematic reviews and Meta‐Analyses (PRISMA) [12].

### 4.1 Inclusion and Exclusion Criteria

This review considered guidelines from national healthcare institutions and from professional healthcare societies that had undergone a systematic literature review and/or a formal consensus process. The verification of correct nasogastric tube placement in adult hospitalised patients was required to be the primary focus of these guidelines or significantly represented within. Only guidelines for adult patients in hospital wards were considered. Guidelines focussed solely on patients under 18, patients in home care, and nursing home residents were excluded. If a guideline addressed these excluded populations but was primarily directed at adult patients in hospital wards, it was included. Reviews, meta‐analyses and experimental studies were excluded. Guidelines published by professionals who did not represent a healthcare organisation or professional healthcare society were also excluded. Guidelines that had not undergone a consensus process and/or systematic literature review, or where the application of these processes was unclear, were excluded.

### 4.2 Search Strategy and Selection of Studies

The systematic literature search was conducted in January 2024 in two medical databases (Medline, CINAHL). In addition, international guideline registers and references of relevant studies were searched. A detailed review of guideline registers was conducted. Each of these registers was systematically searched manually for guidelines relating to the position control of feeding tubes in adult patients in hospitals. A list of all screened guideline registers can be found in the Supporting Information.

The search terms were divided into three components (nasogastric tube, guidelines and placement verification). For the guideline component, a search string developed by Canada's Drug Agency was used that had been formally validated for retrieving clinical practice guidelines [13]. Medline and CINAHL search strings are displayed in the Supporting Information.

The results of the literature search were transferred to EndNote 21, and duplicates were removed. Subsequently, the results were imported into Covidence [14], where a title and abstract screening was performed by two independent persons and analysed in a consensus process. Finally, full‐text screening was performed. In case of conflicts, a third reviewer was consulted.

### 4.3 Quality Assessment

Quality assessment was conducted by four independent reviewers—registered nurses with training in nursing science [3] and medicine [1]—for each of the six included guidelines using the Appraisal of Guidelines for Research & Evaluation II (AGREE‐II tool) [15]. This tool contains six domains – ‘Scope and Purpose’, ‘Stakeholder Involvement’, ‘Rigour of Development’, ‘Clarity of Presentation’, ‘Applicability’, and ‘Editorial Independence’—and an overall Assessment. Each domain is evaluated through multiple items scored on a 7‐point scale, where 1 indicates ‘Strongly Disagree’ and 7 indicates ‘Strongly Agree.’ The scaled domain values (as percentages) are calculated as follows: (totalled domain score − minimum possible domain score): (Maximum possible domain score − minimum possible domain score) [15].

Websites of the publishing organisations were searched manually for additional material, and in two cases, personal contact was made with a representative of the organisation [16, 17].

High‐quality and lower‐quality guidelines were determined on the basis of the AGREE II‐scaled domain ratings. To be classified as a high‐quality guideline, a score of ≥ 60% had to be achieved in the scaled domain assessments in Domain 3 and in two other domains [18].

The use of AGREE‐II domains, ranging from scope and purpose to editorial independence, provided a structured approach to assessing methodological quality. Scaled domain values, their average and standard deviation are displayed in the Supporting Information.

### 4.4 Data Extraction

Data extraction was carried out by two reviewers in consensus. A third person was brought in to oversee this process and compare the extracted data with the sources. Data were extracted in a table including domains of prioritised methods for initial verification, practices and frequency for routine monitoring, and not recommended practices.

## 5 Results

**FIGURE 1:**
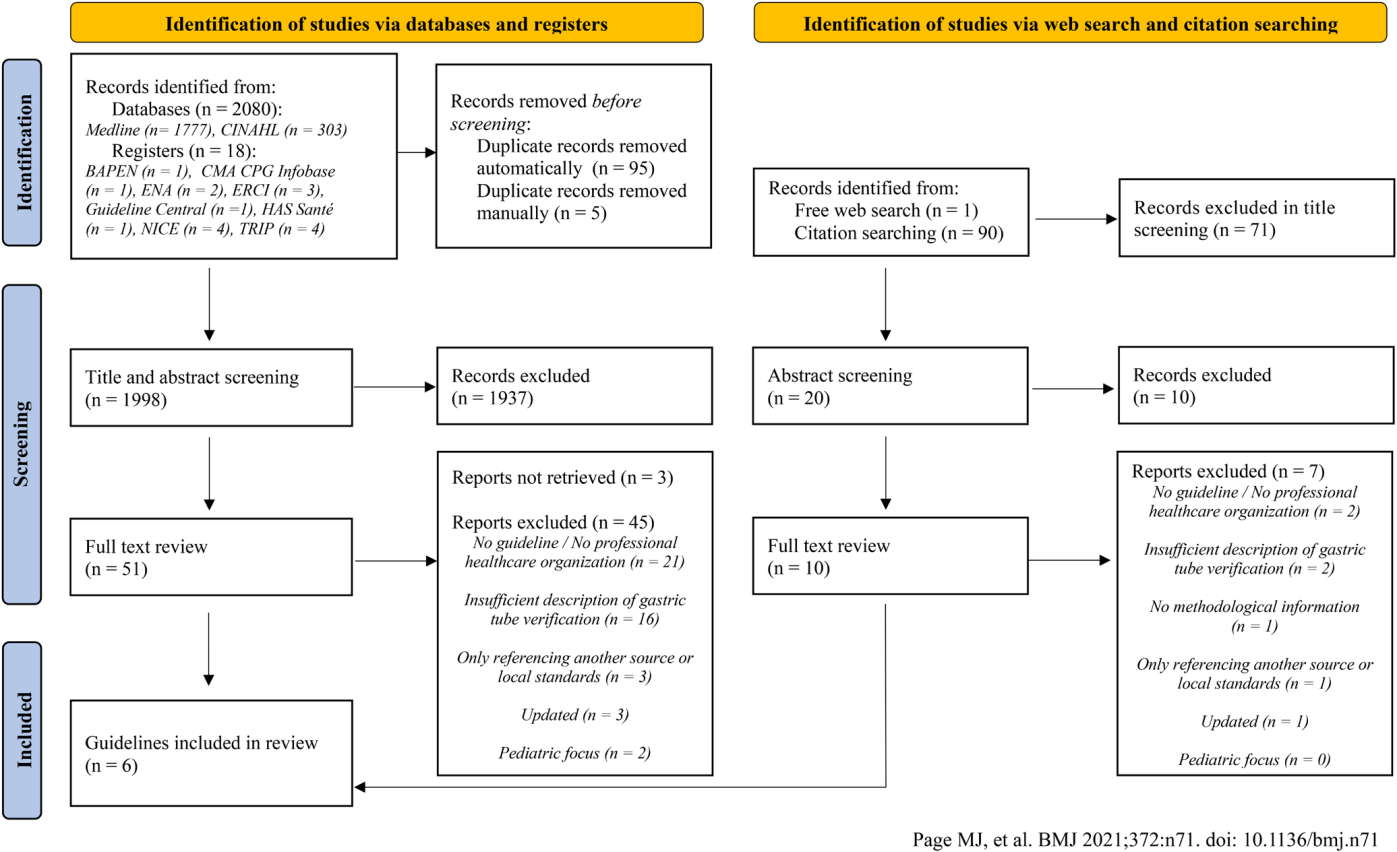
PRISMA flow diagram for systematic reviews.

As displayed in Figure 1 2080 records were identified from Medline (*n* = 1777) and CINAHL (*n* = 303). Eighteen studies were identified in guideline registers, including BAPEN (*n* = 1), CMA CPG Infobase (*n* = 1), ENA (*n* = 2), ERCI (*n* = 3), Guideline Central (*n* = 1), HAS Santé (*n* = 1), NICE (*n* = 4) and TRIP (*n* = 4).

After removing 100 duplicate records, 1998 records proceeded to title and abstract screening. Of these, 1937 records were excluded, leaving 51 reports for full‐text review. During the full‐text review, 48 reports were excluded, including three that were not retrievable in English or German.

Ninety one records were identified through web search (*n* = 1) and citation searching (*n* = 90). Of these, 71 records were excluded in title screening, and 20 records underwent further abstract screening, where 10 records were excluded. Ten reports were retrieved for full‐text review. A list of full‐text reviewed studies with reasons for exclusion can be found in the Supporting Information.

**FIGURE 2:**
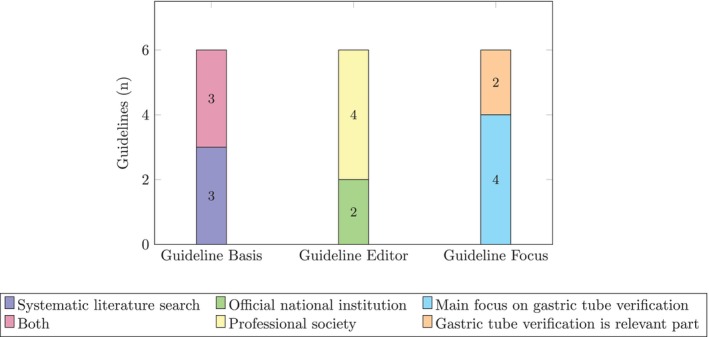
Inclusion characteristics of guidelines.

In total, six guidelines were included in the final review. Three were identified through databases (*n* = 2) and CPG registers (*n* = 1), whereas the other three derived from web searches (*n* = 1) and citation lists (*n* = 2). Four guidelines originated from the United States, one from Singapore, and one from Australia. Figure 2 shows the application of the inclusion criteria for the final set of included guidelines.

## 6 CPG Recommendations

The following results are summarised in the table provided in the Supporting Information. The table includes the country of origin, professional society, as well as prioritised methods for initial verification. In addition, recommended practices and frequency for routine monitoring, practices that are not recommended or for which no recommendation is provided, and the grading of the recommendations are reported.

### 6.1 Bedside Methods During Insertion Process, X‐Ray Before Initial Use

**FIGURE 3:**
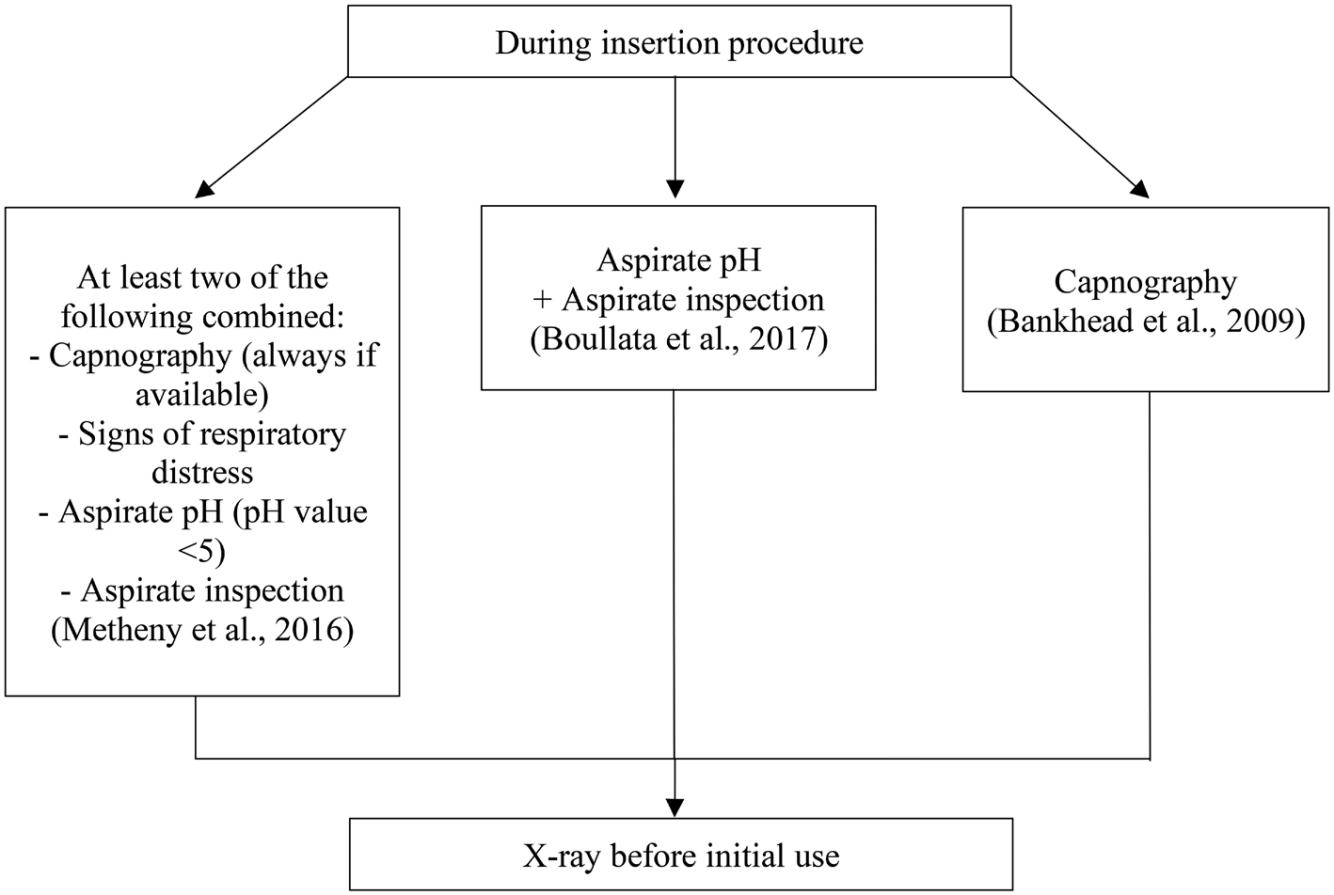
Bedside methods during insertion process.

As seen in Figure 3, three of the six guidelines advise the use of chest radiographs with clear visualisation of the entire gastric tube as the most reliable and safest method of confirming correct tube placement. It is recommended that this procedure should be performed before administering substances such as medication, nutrition or water through the feeding tube [9, 19, 20]. All three guidelines recommending x‐ray confirmation prior to initial use of a gastric tube also emphasise using bedside methods during the insertion process, although recommended bedside methods vary between the guidelines:

The American Association of Critical‐Care Nurses (AACN) [20] advises combining at least two of the following four approaches during the insertion process: observing the patient for respiratory distress, using capnography, measuring the pH of the aspirate (cut‐off value 5), and visually inspecting the aspirate. Bankhead et al. [19] highlight the utility of capnography in identifying if the nasogastric tube has mistakenly entered the trachea during insertion. Boullata et al. [9] advocate for pH measurement and visual inspection of the aspirate throughout the insertion process.

### 6.2 Bedside Methods After Insertion

Two guidelines recommend measuring the initial pH of the tube aspirate to confirm correct placement of a feeding tube, although they differ in the criteria for when an x‐ray is required to ensure correct positioning of the tube. Both agree that an x‐ray should not be performed routinely but should be considered if abnormalities or concerns arise during pH measurement or other aspects of the procedure that risk misplacement of the tube [17, 21].

According to Koh et al. [21], an x‐ray must be conducted if not enough aspirate (< 1 mL) can be obtained from the tube or if the aspirate's pH value exceeds 5.5, and two additional criteria are met. These include the aspirate volume being less than 10 mL or not resembling gastric fluid, the absence of pH‐changing substances in the preceding 24 h, a change in the pH value within the last 24 h, and the absence of a ‘whooshing’ noise when auscultating. If there are signs of tube migration, such as ‘visible coiling in the mouth’, the tube should be reinserted.

According to the New South Wales (NSW) Health guideline [17], an x‐ray should be conducted if no aspirate can be obtained, if the pH value exceeds 5, or if there was difficulty inserting the gastric tube. Additionally, the guideline recommends using x‐ray in cases where the patient has individual risk factors, such as a ‘history of facial fractures’, ‘neurological injury, insult or baseline abnormality’, ‘respiratory concerns’, ‘decreased or absent gag reflex’ or critical illness.

Perry et al. [16] have evaluated various methods for confirming the placement of a feeding tube and found that there is moderate evidence for the use of ultrasound, CO_2_ detection and measuring aspirate pH in combination with other bedside methods. When used alone, there is limited evidence for aspirate pH measurement to confirm correct gastric tube placement. These methods are considered practically relevant for use in the emergency department.

However, Perry et al. do not provide a definitive recommendation regarding the ability of CO_2_ detection, ultrasound or gastric aspirate analysis to detect incorrectly placed gastric tubes. Additionally, they find insufficient evidence for the use of several other methods, including auscultation, electromagnetic devices and aspirate bilirubin testing alone to detect correct tube positioning. They find no evidence that inaccurate gastric tube placement can be detected by transillumination or magnetic detection. They do not indicate whether bedside methods or radiography should be preferred [16].

### 6.3 Monitoring Feeding Tube Position

Five guidelines recommend marking the nasogastric tube at the nasal exit site after confirming correct placement (and documenting the extracorporeal tube length, if measurable). All of these guidelines advise routine monitoring for changes in the external tube length, see length changes as a warning sign for dislocation and recommend action in case of this event [9, 17, 19, 20, 21].

The AACN [20] additionally recommends the routine use of pH measurement, visual inspection (both after feeding is paused for over an hour) and monitoring changes in the aspirate volume. These checks should be performed every 4 h after the start of enteral feeding. Also, they recommend routinely reviewing radiography reports for statements on tube placement.

Koh et al. [21] and NSW Health [17] highlight visible coiling of the tube in the patient's mouth and respiratory distress or discomfort with the tube as key signs of dislocation. Therefore, both guidelines recommend regularly checking for tube coiling in the patient's mouth. In case of visible coiling or in case of tube migration signs, Koh et al. recommend reinsertion of the tube. They further advise measuring a baseline SpO_2_ and aspirate pH before feeding and inspect the visual appearance of the aspirate. Peripheral oxygen saturation should also be measured during and after enteral feeding [21]. The NSW Health guideline gives specific conditions when checks should be carried out: After every shift turnover, before administering substances via the feeding tube, when the patient is repositioned or was transferred to another unit, and after ‘episodes of respiratory distress, vomiting, retching, or coughing’. According to the NSW Health guideline, tube migration signs also include ‘loose tape, visible tube appears long, poor tolerance to feed, reflux of feed into throat, discomfort in the throat or patient pulling at the tube’ [12, 17]. Perry et al. [16] do not provide recommendations for routine checks.

If in doubt of a correct feeding tube position, the guidelines give following recommendations to action if changes in the length of the external part of the tube or other mentioned signs of dislocation are observed [9, 17, 19, 20, 21]: The NSW Health guideline [17] states that either aspirate pH measurement or an x‐ray should be performed. Metheny et al. [20] recommend only a radiographic confirmation in this case. Boullata et al. [9] suggest using both visual inspection of the aspirate and measuring its pH value. Bankhead et al. [19] refer to the use of bedside methods without specifying which particular bedside technique should be used. Both guidelines recommend a radiograph if further doubt on correct tube positioning after these actions remains [9, 19].

### 6.4 pH Threshold

The guidelines differ in their recommendations for the pH threshold to check the correct placement of the feeding tube: Both the NSW Health and AACN guideline recommend a pH below 5 [17, 20], while Koh et al. suggest a pH below 5.5 [21]. Perry et al. [16], Boullata et al. [9] and Bankhead et al. [19] do not make a specific pH recommendation.

### 6.5 Methods Not Recommended

**FIGURE 4:**
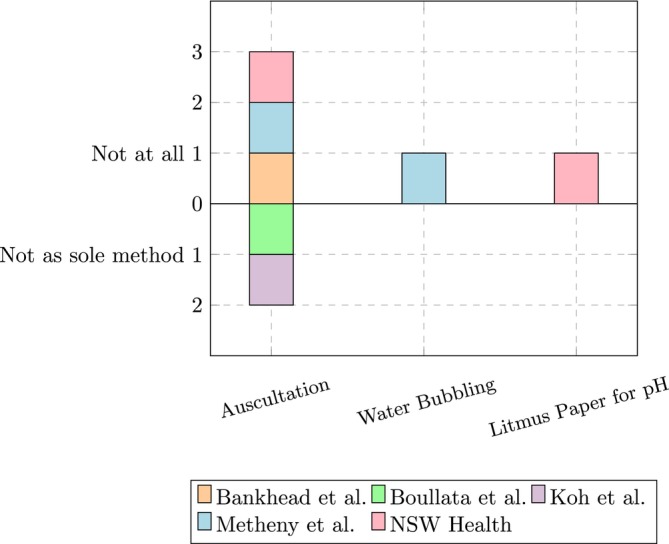
Methods not recommended.

As shown in Figure 4, three guidelines explicitly advise against using the auscultation method in general [17, 19, 20]. The AACN [20] additionally advises against the water bubbling method. The NSW Health guideline additionally advises against using litmus paper to test pH value. Instead, pH indicator sticks with a clear gradation should be used [17]. Two guidelines [9, 21] suggest that auscultation should not be used as the sole verification method, but can be used as a supplementary tool in combination with other techniques. Perry et al. [16] note that there is ‘insufficient or no evidence to make a recommendation’ for using the auscultation method, electromagnetic devices, or bilirubin testing of gastric aspirates alone to verify correct gastric tube placement. Similarly, they report ‘insufficient or no evidence to make a recommendation’ for detecting tube misplacement using ultrasound, CO_2_ detection, transillumination, or magnetic detection.

### 6.6 Methodological Strengths and Weaknesses

Only one of the six guidelines, Perry et al. [16], met the conditions for high quality, with scoring ≥ 60% in Domain 3 and in two other domains. They provided supporting material detailing the guideline development process of the Emergency Nurses Association, an international organisation of emergency nursing professionals. However, they give no specific description of the process of this particular guideline.

**Domain 1** (‘Scope and Purpose’) and **Domain 4** (‘Clarity of Presentation’) were the highest scoring domains, both averaging above 60%. In almost all guidelines, the objectives and specific health issues were clarified, the target groups were clearly defined and the recommendations were precise and easy to find. Significant deficiencies were identified specifically in four key AGREE‐II domains.

**Domain 2:** *Stakeholder Involvement*—Many guidelines lacked diverse representation from stakeholders [19] or did not give any information which persons were participating in guideline development [17, 20], weakening the inclusiveness and relevance of their recommendations. Without input from all stakeholders, the guidelines risk excluding important perspectives for applicability and acceptance of their recommendations.

**Domain 3:** *Rigour of Development*—The reviewed guidelines often failed to document critical elements of their development such as search terms, databases searched, inclusion/exclusion criteria and evidence levels of their recommendations [9, 17, 20, 21]. These omissions contributed to lower scores and raised concerns about the reliability and validity of the recommendations. Only one guideline met the criterion of scoring ≥ 60% in this crucial domain, illustrating a lack of transparency or adherence concerning systematic processes and evidence rigour.

**Domain 5:** *Applicability*—There was a lack of detailed strategies on overcoming implementation barriers, cost implications, or tools to aid the uptake of recommendations into clinical settings [9, 16, 17, 19, 20]. Without this information, the utility of the guidelines for healthcare professionals is limited as they may be left without adequate support to effectively translate recommendations into practice.

**Domain 6:** *Editorial Independence*—In all guidelines, funding sources were disclosed insufficiently, and/or conflicts of interest were not transparently reported [9, 16, 17, 19, 20, 21]. This lack of transparency makes it challenging to determine whether recommendations were free from potential bias or external influence, ultimately compromising the credibility of the guidelines.

## 7 Discussion

This systematic review provides critical insights into the current landscape of international guidelines for gastric tube placement verification in adult patients in hospitals, highlighting significant discrepancies in recommended practices for almost all aspects of gastric tube position verification. A considerable variability in methodological transparency and inconsistency in the primary sources' evidence levels was observed across guidelines. In some instances, the rationale behind recommendations, their level of evidence, or even the authors of the guideline remained unclear. Guidelines differed in evaluating evidence strength, number and evidence requirements of primary sources used for recommendation, resulting in differing conclusions. This underscores the need for more high‐quality primary research to establish evidence‐based best practices, greater international standardisation to minimise clinical ambiguity, and ensuring the highest scientific standards in the development of practice‐based guidelines through methodological rigour.

Five guidelines explicitly recommend not to use auscultation for tube placement verification. Although one guideline does not advise against auscultation, it also does not recommend this method and its use had been discouraged in previous editions of the same guideline [16]. The rationale behind this change of recommendation was not fully comprehensible. This highlights the importance of de‐implementation of practices not supported by evidence [22, 23].

This review's findings align with and build on those of Metheny et al. [10] confirming that x‐rays remain the gold standard for verification of gastric tube placement, especially in high‐risk patients, though not always recommended as a first‐line method. The review highlights guidelines recommending bedside methods to complement x‐rays, though these methods vary.

This review identified significant variability in non‐radiological methods, which is in line with the review by Metheny et al. [10], that identified pH testing as the most preferred bedside method, with varying pH cut‐offs. In contrast, our review notes that while some guidelines prioritise pH testing, there is still no consensus on the optimal threshold.

Auscultation is currently not recommended, but assessing aspirate is more accepted in guidelines than before [10]. Observation for signs of respiratory distress remains useful in detecting tube misplacement. This review stresses the importance of routine checks, with variations in guideline frequency and methods.

## 8 Strengths and Limitations

The methodological rigour in identifying and assessing guidelines and their quality is a clear strength of this work. A systematic literature search was carried out in order to cover all relevant sources and create a solid basis for the analysis with blinding of reviewers applied on all levels of the process. Guidelines were evaluated by experts with relevant experience using the AGREE‐II tool to provide a robust assessment of guideline quality and applicability. In addition, the process was strictly based on the PRISMA criteria, which ensure a high level of transparency and reproducibility of the methodology, underlining the scientific quality and validity of the work.

Limitations of this systematic review should also be noted. First, the scope was limited to guidelines from national healthcare institutions and medical societies that are primarily aimed at adult patients in hospitals. This exclusion of paediatric, non‐hospital‐specific guidelines may limit the applicability of the results to other patient groups where there are also risks associated with feeding tube placement. Second, despite a systematic search strategy in databases and guideline registers, it is possible that relevant guidelines were overlooked. Limiting the search to CINAHL and Medline and to only German and English may have resulted in missing relevant guidelines from less accessible registers or non‐English and non‐German publications. In addition, the restriction to guidelines that had undergone a systematic literature search and/or a formal consensus process further limited the choice of guidelines, potentially excluding less thoroughly reviewed practices. The small number and the heterogeneity of the included guidelines limit the applicability of the review results. The six analysed guidelines show great variation in methodological quality or transparency and preferred verification methods. These differences may be based on specific contexts of scientific foundation and the healthcare contexts in which the guidelines were developed. The marked heterogeneity complicated the synthesis and presentation of recommendations and highlights the need for evidence‐based international standards that would enable safe verification of gastric tube placement across different healthcare systems. Also, a larger body of guidelines based on high‐quality primary sources would be necessary to establish evidence‐based, universally applicable recommendations.

## 9 Recommendations for Practice and Further Research

Aspirate pH measurement is recommended as a safe alternative to radiographic confirmation for verifying the position of a gastric tube. Once the position of the tube is confirmed, it is recommended to mark the tube exit point and routinely monitor the external tube length to detect any potential displacements. Scheduled checks are recommended to ensure consistent monitoring.

The auscultation method is not recommended and should not be used as sole verification technique. If there is any doubt about the correct gastric tube position, an x‐ray is recommended. Comparative studies are needed to evaluate the diagnostic accuracy and the effectiveness of bedside verification methods in reducing reliance on x‐ray and help determine accurate tube position where x‐ray is not available. Research into novel technologies, for example, electromagnetic devices and advanced imaging techniques in ultrasound, could enhance accuracy and reduce reliance on x‐ray. Studies should be conducted that establish a link between review practices and patient safety outcomes, with a focus on rates of complications and misplacements. Future guidelines must use high‐quality primary studies and focus on methodological transparency and quality, explaining the level of evidence and the systematic evaluation processes behind the recommendations.

## 10 Conclusion

The aim of this systematic review was to summarise and critically evaluate current international guidelines on placement of gastric tubes in adult hospital patients, focussing on recommended practices and methodological quality. Identifying areas of consensus, variability and methodological weaknesses, the review aimed to highlight gaps in the evidence base and serve as a basis for future research and guideline development to improve patient safety.

The auscultation method is considered unreliable for tube placement confirmation. Aspirate pH is considered a safe alternative to radiographic imaging for initial verification. If uncertainty exists about tube positioning, radiographic confirmation is recommended. There is agreement that once the correct placement has been confirmed, the exit site of the gastric tube should be clearly marked and the external length should be regularly recorded and monitored to identify possible dislodgement. Regular position checks are advised.

Guideline recommendations are heterogeneous, not only in their recommendations but also in their methodological quality. There is considerable variability in how different guidelines assess verification methods, frequency of checks and protocols for suspected dislodgement. While there is some consensus, great variation persists. This highlights the need for ongoing high‐quality research and international standardisation efforts to ensure safe and effective patient care.

## Funding

The authors have nothing to report.

## Ethics Statement

The authors have nothing to report.

## Consent

The authors have nothing to report.

## Conflicts of Interest

The authors declare no conflicts of interest.

## Supporting information


**Table S1:** Full‐text reviewed studies.
**Table S2:** Scaled domain scores of the guidelines.
**Table S3:** CPG recommendations.
**Table S4:** Guideline registers screened.
**Figure S1:** Average scaled domain scores with SD.

## Data Availability

Data sharing not applicable to this article as no datasets were generated or analysed during the current study.

## References

[1] F. Streibert , C. Bernhardt , P. Simon , P. Hilbert‐Carius , and H. Wrigge , “Sichere Lagekontrolle von Magensonden: ein oft Unterschätztes Thema zur Vermeidung potenziell schwerwiegender Komplikationen,” Anaesthesiology 72, no. 1 (2023): 57–62. 10.1007/s00101-022-01218-4 36416892

[2] Thiemes Pflege: Das Lehrbuch für Pflegende in Ausbildung, 15th ed. (Thieme, 2021).

[3] A. P. G. Motta , M. C. G. Rigobello , R. C. D. C. P. Silveira , and F. R. E. Gimenes , “Nasogastric/Nasoenteric Tube‐Related Adverse Events: An Integrative Review,” Revista Latino‐Americana de Enfermagem 29 (2021): e3400. 33439952 10.1590/1518-8345.3355.3400 PMC7798396

[4] A. S. Hanna , C. R. Grindle , A. A. Patel , M. R. Rosen , and J. J. Evans , “Inadvertent Insertion of Nasogastric Tube Into the Brain Stem and Spinal Cord After Endoscopic Skull Base Surgery,” American Journal of Otolaryngology 33, no. 1 (2012): 178–180. 21715048 10.1016/j.amjoto.2011.04.001

[5] N. A. Metheny , K. L. Meert , and R. E. Clouse , “Complications Related to Feeding Tube Placement,” Current Opinion in Gastroenterology 23, no. 2 (2007): 178–182. 17268247 10.1097/MOG.0b013e3280287a0f

[6] D. A. Sparks , D. M. Chase , L. M. Coughlin , and E. Perry , “Pulmonary Complications of 9931 Narrow‐Bore Nasoenteric Tubes During Blind Placement: A Critical Review,” Journal of Parenteral and Enteral Nutrition 35, no. 5 (2011): 625–629. 21799186 10.1177/0148607111413898

[7] M. L. C. Ellett , “What Is Known About Methods of Correctly Placing Gastric Tubes in Adults and Children,” Gastroenterology Nursing 27, no. 6 (2004): 253–259. 15632757 10.1097/00001610-200411000-00002

[8] S. A. Milsom , J. A. Sweeting , H. Sheahan , E. Haemmerle , and J. A. Windsor , “Naso‐Enteric Tube Placement: A Review of Methods to Confirm Tip Location, Global Applicability and Requirements,” World Journal of Surgery 39, no. 9 (2015): 2243–2252. 25900711 10.1007/s00268-015-3077-6

[9] J. I. Boullata , A. L. Carrera , L. Harvey , A. A. Escuro , L. Hudson , and A. Mays , “ASPEN Safe Practices for Enteral Nutrition Therapy,” Journal of Parenteral and Enteral Nutrition 41, no. 1 (2017): 15–103. 27815525 10.1177/0148607116673053

[10] N. A. Metheny , M. M. Krieger , F. Healey , and K. L. Meert , “A Review of Guidelines to Distinguish Between Gastric and Pulmonary Placement of Nasogastric Tubes,” Heart & Lung 48, no. 3 (2019): 226–235. 30665700 10.1016/j.hrtlng.2019.01.003

[11] C. Baving , M. Grebe , M. Dichter , S. Koepke , and PROSPERO , “Guidelines for Verification of Gastric Tubes Location in Adult Hospitalized Patients ‐ A Systematic Review,” (2024), https://www.crd.york.ac.uk/PROSPERO/view/CRD42024537473.10.1111/nicc.7046641913447

[12] M. J. Page , J. E. McKenzie , P. M. Bossuyt , et al., “The PRISMA 2020 Statement: An Updated Guideline for Reporting Systematic Reviews,” BMJ 372 (2021): n71. 33782057 10.1136/bmj.n71 PMC8005924

[13] “CADTH Search Filters Database [Internet],” (2024), Guidelines ‐ Broad – CINAHL, https://searchfilters.cadth.ca/link/74.

[14] Veritas Health Innovation , “Covidence Systematic Review Software [Internet],” (2024), Melbourne, Australia, https://www.covidence.org.

[15] M. C. Brouwers , M. E. Kho , G. P. Browman , J. S. Burgers , F. Cluzeau , and G. Feder , “AGREE II: Advancing Guideline Development, Reporting and Evaluation in Health Care,” Canadian Medical Association Journal 182, no. 18 (2010): E839–E842. 20603348 10.1503/cmaj.090449 PMC3001530

[16] A. Perry , J. Kaiser , K. Kruger , A. E. Horigan , J. Y. Bradford , and A. Camarda , “ENA Clinical Practice Guideline Synopsis: Gastric Tube Placement Verification,” Journal of Emergency Nursing 50, no. 2 (2024): 301–304. 38453344 10.1016/j.jen.2023.09.001

[17] NSW Health Government , “Insertion and Management of Nasogastric and Orogastric Tubes in Adults,” (2023), St. Leonards NSW (AU).

[18] S. Bargeri , V. Iannicelli , G. Castellini , M. Cinquini , and S. Gianola , “AGREE II Appraisals of Clinical Practice Guidelines in Rehabilitation Showed Poor Reporting and Moderate Variability in Quality Ratings When Users Apply Different Cuff‐Offs: A Methodological Study,” Journal of Clinical Epidemiology 139 (2021): 222–231. 34437947 10.1016/j.jclinepi.2021.08.021

[19] R. Bankhead , J. Boullata , S. Brantley , et al., “A.S.P.E.N. Enteral Nutrition Practice Recommendations,” Journal of Parenteral and Enteral Nutrition 33, no. 2 (2009): 122–167. 19171692 10.1177/0148607108330314

[20] N. A. Metheny , “Initial and Ongoing Verification of Feeding Tube Placement in Adults (Applies to Blind Insertions and Placements With an Electromagnetic Device),” Critical Care Nurse 36, no. 2 (2016): e8–e13. 10.4037/ccn2016141 27037348

[21] P. Koh , T. S. Chin , A. Lee , P. Tan , T. P. Janet , and P. Lai , “National Guidelines on Nursing Management of Nasogastric Tube in Adult Patients,” (2022).

[22] L. N. Tume , L. Kenworthy , E. C. Alexander , et al., “Identifying Low Value Care Practices in uk Paediatric Intensive Care Units in 2025: A Delphi Study,” Nursing in Critical Care 30, no. 6 (2025): e70235. 41208231 10.1111/nicc.70235 PMC12598117

[23] L. N. Tume and L. M. Aitken , “De‐Implementation of Low Value Clinical Practices Is Essential for Critical Care Nurses,” Nursing in Critical Care 29, no. 2 (2024): 244–245. 38375598 10.1111/nicc.13028

