# Supplementary material for: Guidelines for Verification of Gastric Tube Location in Adult Hospitalised Patients: A Systematic Review
Source: Nurs Crit Care. 2026 Mar 30;31(3):e70466. doi: 10.1111/nicc.70466 (PMC13036308; doi:10.1111/nicc.70466)
Supplement: Supplementary file 1 — Table S1: Full‐text reviewed studies. Table S2: Scaled domain scores of the guidelines. Table S3: CPG recommendations. Table S4: Guideline registers screened. Figure S1: Average scaled domain scores with SD. [file NICC-31-0-s001.docx]

**Appendix**

**Full-text reviewed Studies (n = 61)**

| Year, Country | Organization | Author(s) | Title | Inclusion | Exclusion reason(s) |
| --- | --- | --- | --- | --- | --- |
| 1994, US | American Association of Critical-Care Nurses | Rakel et al. | Nasogastric and nasointestinal feeding tube placement: an integrative review of research | No | No guideline |
| 2009, US | American Association of Critical-Care Nurses | Metheny, N. | Verification of Feeding Tube Placement (blindly inserted) | No | Updated |
| 2016, US | American Association of Critical-Care Nurses | Metheny et al. | Initial and Ongoing Verification of Feeding Tube Placement in Adults | Yes |  |
| 2022, US | American Association of Critical-Care Nurses | Bloom et al. | Placement of Nasogastric Feeding Tube and Postinsertion Care Review | No | No guideline |
| 2016, US | American College of Gastroenterology | McClave et al. | Nutrition Therapy in the Adult Hospitalized Patient | No | Insufficient description of gastric tube verification |
| 1995, US | American Gastroenterological Association | Not published | American Gastroenterological Association Medical Position Statement: guidelines for the use of enteral nutrition | No | Insufficient description of gastric tube verification |
| 2011, US | American Gastroenterological Association, Society of Interventional Radiology | Itkin et al. | Multidisciplinary Practical Guidelines for Gastrointestinal Access for Enteral Nutrition And Decompression | No | Insufficient description of gastric tube verification |
| 2000, FR | Agence Nationale d’Accréditation et d’Évaluation en Santé | Leverve et al. | Care and Monitoring of Enteral Access for Enteral Nutrition in Adults in Hospital and at Home | No | Insufficient description of gastric tube verification |
| 2002, US | American Society  for Parenteral and Enteral Nutrition | August et al. | Guidelines for the Use of Parenteral and Enteral Nutrition in Adult and Pediatric Patients | No | Insufficient description of gastric tube verification |
| 2009, US | American Society  for Parenteral and Enteral Nutrition | Bankhead et al. | ASPEN Enteral nutrition practice recommendations | Yes |  |
| 2016, US | American Society  for Parenteral and Enteral Nutrition | Boullata et al. | ASPEN Safe Practices for Enteral Nutrition Therapy | Yes |  |
| 2016, US | American Society  for Parenteral and Enteral Nutrition, Society of Critical Care Medicine | McClave et al. | ASPEN Guidelines for the Provision and Assessment of Nutrition Support Therapy in the Adult Critically Ill Patient | No | Insufficient description of gastric tube verification |
| 2020, US | American  Society for Parenteral and Enteral Nutrition, Society of Critical Care Medicine | Martindale et al. | Nutrition Therapy in the Patient with COVID-19 Disease Requiring ICU Care | No | Insufficient description of gastric tube verification |
| 2021, US | American Society  for Parenteral and Enteral Nutrition | Powers et al. | Development of a Competency Model for Placement and Verification of Nasogastric and Nasoenteric Feeding Tubes for Adult Hospitalized Patients | No | Insufficient description of gastric tube verification |
| 2022, US | American Society for Parenteral and Enteral Nutrition | Cattani et al. | A systematic review on the agreement between clinical practice guidelines regarding the steps of the nutrition care process of adult patients who are critically ill | No | No guideline |
| 2003, UK | British Association for Parenteral and Enteral Nutrition | Stroud et al. | Guidelines for enteral feeding in adult hospital patients | No | Only referencing another source or local standards |
| 2020, UK | British Association for Parenteral and Enteral Nutrition | BAPEN’s Nasogastric tube safety Special Interest Group | Enteral tube feeding safety in COVID-19 patients | No | Only referencing another source or local standards |
| 2020, UK | British Association for Parenteral and Enteral Nutrition | Jones et al. | A Position Paper on Nasogastric Tube Safety | No | No guideline |
| 2011, CA | BC Cancer Agency | Orphanidou et al. | Prophylactic feeding tubes for patients with locally advanced head-and-neck cancer undergoing combined chemotherapy and radiotherapy—systematic review and recommendations for clinical practice | No | No guideline |
| 2020,  UK | British Dietetic Association | Hardy et al. | BDA Critical Care Specialist Group COVID-19 Best Practice Guidance: Feeding Patients on Critical Care Units in the Prone Position (awake and sedated). | No | Insufficient description of gastric tube verification |
| 2003, CA | The Canadian Critical Care Society, Canadian Institute of Health Research, The Canadian Society for Clinical Nutrition | Heyland et al. | Canadian clinical practice guidelines for nutrition support in mechanically ventilated, critically ill adult patients | No | Insufficient description of gastric tube verification |
| 2015, CN | Chinese Medical Association's Nutrition Support | Wei et al. | Guidelines for parenteral and enteral nutrition support  in geriatric patients in China | No | Insufficient description of gastric tube verification |
| 2023, CN | Chinese Society of Parenteral and Enteral Nutrition | Hua et al. | Guideline for clinical application of parenteral and enteral nutrition in adults patients in China | No | Not retrieved in English or German language |
| 2004, UK | Clinical Resource Efficiency Support Team | Holmes et al. | Guidelines for the Management of Enteral Tube Feeding in Adults | No | Only referencing another source or local standards |
| 2023, UK | The College of Radiographers | Hamdaoui et al. | A scoping review of clinical practices and adherence to UK national guidance related to the placement and position confirmation of adult nasogastric feeding tubes | No | No guideline |
| 2018, USA | Emergency Nurses Association | Killian et al. | Clinical Practice Guideline: Gastric Tube Placement Verification | No | Updated |
| 2024, USA | Emergency Nurses Association | Perry et al. | ENA Clinical Practice Guideline Synopsis: Gastric Tube Placement Verification | Yes |  |
| 2006, EU | The European Society for Clinical Nutrition and Metabolism | Lochs et al. | ESPEN guidelines on enteral nutrition: gastroenterology | No | Insufficient description of gastric tube verification |
| 2009, EU | The European Society for Clinical Nutrition and Metabolism | Bozzetti et al. | The ESPEN clinical practice guidelines on Parenteral Nutrition: Present status and perspectives for future research | No | No guideline |
| 2018, EU | The European Society for Clinical Nutrition and Metabolism | Burgos et al. | ESPEN guideline clinical nutrition in neurology | No | Insufficient description of gastric tube verification |
| 2022, EU | The European Society for Clinical Nutrition and Metabolism | Bischoff et al. | ESPEN guideline: Clinical nutrition in inflammatory bowel disease | No | Insufficient description of gastric tube verification |
| 2021, EU | European Society of Gastrointestinal Endoscopy | Gkolfakis et al. | Endoscopic management of enteral tubes in adult patients - Part 2: Peri- and post-procedural management. | No | Insufficient description of gastric tube verification |
| 2010, SG | Ministry of Health Singapore | Tan et al. | Nursing Management of Nasogastric Tube Feeding in Adult Patients | No | Updated |
| 2022, SG | Ministry of Health Singapore | Koh et al. | National Guidelines on Nursing Management of Nasogastric Tube in Adult Patients | Yes |  |
| 2006, UK | National Collaborating Centre for Acute Care | Stroud et al. | Nutrition Support for Adults Oral Nutrition Support, Enteral Tube Feeding and Parenteral Nutrition | No | Only referencing another source or local standards |
| 2011, UK | National Health Service | Not published | Reducing the harm caused by misplaced nasogastric feeding tubes in adults, children and infants | No | No guideline |
| 2016, UK | National Health Service | Not published | Resource set. Initial placement checks for nasogastric and orogastric tubes | No | No guideline |
| 2021, UK | National Health Service | McGavin et al. | Radiological confirmation of correct placement of nasogastric tubes in Adults, Children and Neonates for feeding | No | No guideline |
| 2023, UK | National Health Service | Not published | Adult Nasogastric Tube Insertion Procedure and Management Policy | No | No guideline |
| 2013, UK | National Institute for Health and Care Excellence | Elia et al. | Nutrition support in adults | No | Insufficient description of gastric tube verification |
| 2016, UK | National Institute for Health and Care Excellence | Chalkidou et al. | CORTRAK 2 Enteral Access System for placing nasoenteral feeding tubes | No | Insufficient description of gastric tube verification |
| 2019, UK | National Institute for Health and Care Excellence | Not published | Assessing and monitoring complications and comorbidities: feeding and nutritional problems | No | No guideline |
| 2012, UK | National Nurses Nutrition Group | Earley et al. | Good Practice Guidance Safe Insertion of Nasogastric (NG) Feeding Tubes in Adults - not ongoing care | No | Updated |
| 2016, UK | National Nurses Nutrition Group | Anderson et al. | Good Practice Guideline. Safe Insertion and Ongoing Care of Nasogastric (NG) Feeding Tubes in Adults | No | No methodological information |
| 2011, SG | National University Health System | Tho et al. | Implementation of the evidence review on best practice for confirming the correct placement of nasogastric tube in patients in an acute care hospital | No | No guideline |
| 2023, AU | New South Wales Ministry of Health | Not published | Insertion and Management of Nasogastric and Orogastric Tubes in Adults | Yes |  |
| 2013, UK | The Royal College of Radiologists | Law et al. | Avoiding never events: Improving nasogastric intubation practice and standards | No | No guideline |
| 2011, SP | Spanish Society of Intensive Care Medicine and Coronary Units,  Spanish Society of Parenteral and Enteral Nutrition | Fernández-Ortega et al. | Guidelines for specialized nutritional and metabolic support in the critically ill patient | No | Insufficient description of gastric tube verification |
| 2009, AU | No affiliation | Peter et al. | Development of a clinical practice guideline for testing nasogastric tube placement | No | Pediatric focus |
| 2019, US | No affiliation | Metheny et al. | A review of guidelines to distinguish between gastric and pulmonary placement of nasogastric tubes | No | No guideline |
| 1981, US | No affiliation | Gordon, A. | Enteral nutritional support  Guidelines for feeding tube selection and placement | No | No guideline |
| 2012, US | No affiliation | Stepter, C. | Maintaining Placement of Temporary Enteral Feeding Tubes in Adults: A Critical Appraisal of the Evidence | No | No guideline |
| 2019, TW | No affiliation | Yang et al. | The Feasibility Study of a Revised Standard Care Procedure on the Capacity of Nasogastric Tube Placement Verification Among Critical Care Nurses | No | No guideline |
| 2016, UK | No affiliation | Best, C. | How to insert a nasogastric tube and check gastric position at the bedside | No | No professional healthcare institution |
| 2002, UK | No affiliation | Riley, M. | Establishing nutritional guidelines for critically ill patients: Part 1 | No | No guideline |
| 2002, UK | No affiliation | Riley, M. | Establishing nutritional guidelines for critically ill patients: Part 2 | No | No guideline |
| 2007, US | No affiliation | Bourgault et al. | Development of evidence-based guidelines and critical care nurses' knowledge of enteral feeding | No | No guideline |
| 2005, UK | No affiliation | Khair, J. | Guidelines for testing the placing of nasogastric tubes | No | Pediatric focus |
| 2011, US | No affiliation | Tripathi et al. | Best Practices to Verify Ongoing Placement of NG or OG Tube After Initial X-ray Confirmation | No | No guideline |
| 2001, JP | Unknown | Usui et al. | Routes of enteral feeding and guidelines for applications in enteral nutrition | No | Not retrieved in English or German language |
| 2021, CN | Unknown | Mi et al. | Expert consensus on prevention and management of enteral nutrition therapy complications for critically ill patients in China | No | Not retrieved in English or German language |

Table 1: Full-text reviewed studies

**AGREE-II: Scaled domain scores**

Table 2: Scaled domain scores of the guidelines (1)

Figure 1: Average scaled domain scores with SD (1)

**CPG recommendations**

| **Guideline**  **Country**  **Professional Society** | **Prioritized methods for initial verification** | **Practices and frequency for routine monitoring** | **Not recommended practices or no recommendation in general** | **Grading** |
| --- | --- | --- | --- | --- |
| Bankhead et al. 2009,  USA,  American Society for Parenteral and Enteral Nutrition (A.S.P.E.N.) (2) | - X-ray before initial use (Level B) - Capnography during insertion  (Level B) | - Monitor extracorporeal tube length (mark exit site at time of first radiograph)  (Level B) - In doubt other bed-side methods  (Level B) - In further doubt x-ray (Level B) | - Auscultation in general  (Level A) | Level A:  good research based evidence  Level B:  Fair research-based evidence  Level C:  expert opinion, editorial consensus |
| Boullata et al. 2017,  USA,  American Society for Parenteral and Enteral Nutrition (A.S.P.E.N.) (3) | - X-ray before initial use - Aspirate pH and visual inspection during insertion | - Monitor extracorporeal tube length (at time of initial placement, document either incremental marking or external length) - In doubt aspirate pH, visual inspection - In further doubt x-ray | - Auscultation as sole method | No grading of recommendations used |
| Koh et al.  2022,  Singapore,  Ministry of Health Singapore (4) | **First approach:**   - Visually examination of the aspirate before pH testing (gastric, intestinal or respiratory content) - At least 1 ml aspirate for purpose of pH testing - pH ≤ 5.5 safe to proceed with feeding - pH > 5.5 may not be in the stomach. Visual checks (no visible misplacement, no coiling of the NGT) before decision criteria have been fulfilled   Decision criteria at least 2 or more of the following:   - Aspirate >10 ml and contains gastric content - “pH altering medication” (last 24h) - “Same pH or lower” (last 24 h) - “Loud and clear “whooshing” sound during auscultation” (in conjunction with the first approach and others (clinical judgement, nature of aspirate, length of NGT, no coiling etc.)   **If first approach is not successful:**   - X-ray | - In doubt reinsertion   Before feeding:   - Monitor extracorporeal tube length - Check for tube coiling in patient’s mouth - Baseline SpO_2_ - Aspirate pH + visual inspection   During, after feeding: SpO_2_ | - Auscultation as sole method | No grading of recommendations used |
| Metheny et al. 2016,  USA,  American Association of Critical-Care Nurses (AACN) (5) | - X-ray before initial use (Level A) - Two or more methods combined during insertion (Level B):   - Capnography   - Aspirate pH   - Aspirate inspection   - Check for signs of respiratory distress | Check tube location every 4 hours after start of feeding (Level B):   - Monitor extracorporeal tube length - Review radiographic reports - In doubt x-ray (mark exit site) - Volume changes - pH (Feeding pause > 1h) - Visual inspection of aspirate’s appearance (Feeding pause > 1h) | - Auscultation in general  (Level B) - Water bubbling  (Level B) | **Level A** Meta-analysis of quantitative studies or metasynthesis of qualitative studies with results that consistently support a specific action, intervention, or treatment (including systematic review of randomized controlled trials)  **Level B** Well-designed, controlled studies with results that consistently support a specific action, intervention, or treatment  **Level C** Qualitative studies, descriptive or correlational studies, integrative reviews, systematic reviews, or randomized controlled trials with inconsistent results  **Level D** Peer-reviewed professional and organizational standards with the support of clinical study recommendations  **Level E** Multiple case reports, theory-based evidence from expert opinions, or peer-reviewed professional organizational standards without clinical studies to support recommendations  **Level M** Manufacturer’s recommendations only |
| NSW Health  2023,  Australia,  New South Wales Government (6) | Non-radiological confirmation:   - Aspirate pH testing   Radiological confirmation:   - X-ray if :   - No aspirate   - Aspirate pH > 5   - Risk factors of misplacement   - Difficulties during insertion | - In doubt aspirate pH or x-ray   After every shift turnover, repositioning of patient, transfer to another unit. “episodes of respiratory distress, vomiting, retching, or coughing” and before administering substances:   - Monitor extracorporeal tube length (exit-point mark, note and document nasogastric tube exit point to proximal end, compare to initial measurement) - Check for tube coiling in patient’s mouth | - Auscultation in general - Litmus paper for pH testing | No grading of recommendations used |
| Perry et al.  2024,  USA,  Emergency Nurses Association (ENA) (7) | Moderate evidence (Level B):   - Aspirate pH + other bed-side methods - Ultrasound - CO_2_-detection   Limited evidence (Level C):   - Aspirate pH alone |  | Insufficient or no evidence to make a recommendation for verification (Level INE):   - Electromagnetic devices - Auscultation - Bilirubin testing   Insufficient evidence to make a recommendation for/against detecting misplacement (Level INE):   - Ultrasound - Capnography - Gastric aspirate   No evidence (Level INE):   - Transillumination - Magnetic detection | Level A: Consistent and good quality of evidence*  Level B: Minor inconsistencies in quality of evidence*  Level C: Limited or low-quality patient-oriented evidence*  Level NR: Not recommended based upon current evidence.  Level INE: Insufficient or no evidence upon which to make a recommendation.  *Relevance and applicability in emergency nursing practice |

Table 3: CPG recommendations

**Medline search string**

("Clinical protocols"[MeSH Terms] OR "Consensus"[MeSH Terms] OR "Consensus development conferences as topic"[MeSH Terms] OR "Critical pathways"[MeSH Terms] OR "Guidelines as topic"[MeSH Terms:noexp] OR "Practice guidelines as topic"[MeSH Terms] OR "Health planning guidelines"[MeSH Terms] OR "Clinical Decision Rules"[MeSH Terms] OR "guideline"[Publication Type] OR "practice guideline"[Publication Type] OR "consensus development conference"[Publication Type] OR "consensus development conference, nih"[Publication Type] OR "position statement*"[Title/Abstract] OR "policy statement*"[Title/Abstract] OR "practice parameter*"[Title/Abstract] OR "best practice*"[Title/Abstract] OR "standards"[Title] OR "guideline"[Title] OR "guidelines"[Title] OR "standards"[Other Term] OR "guideline"[Other Term] OR "guidelines"[Other Term] OR "guideline*"[Author - Corporate] OR "standards"[Author - Corporate] OR "consensus*"[Author - Corporate] OR "recommendat*"[Author - Corporate] OR "practice guideline*"[Title/Abstract] OR "treatment guideline*"[Title/Abstract] OR "CPG"[Title/Abstract] OR "CPGs"[Title/Abstract] OR "clinical guideline*"[Title/Abstract] OR "guideline recommendation*"[Title/Abstract] OR "consensus*"[Title/Abstract] OR (("critical"[Title/Abstract] OR "clinical"[Title/Abstract] OR "practice"[Title/Abstract]) AND ("position check"[Title/Abstract] OR "Verification"[Title/Abstract] OR "Confirmation"[Title/Abstract] OR "Determination"[Title/Abstract] OR "Control"[Title/Abstract] OR "Application"[Title/Abstract] OR "tube placement determination"[Title/Abstract]))) AND ("nasogastric tube"[Title/Abstract] OR "enteral feeding tube"[Title/Abstract] OR "nasoenteral tube"[Title/Abstract] OR "gastric tube"[Title/Abstract] OR "stomach tube"[Title/Abstract] OR "enteral tube"[Title/Abstract] OR "Feeding Tubes"[Title/Abstract] OR "intubation, gastrointestinal"[MeSH Terms] OR "gastrointestinal intubation"[Title/Abstract] OR "NG tube"[Title/Abstract] OR "OG tube"[Title/Abstract] OR "gastric lavage"[MeSH Terms] OR "enteral nutrition/adverse effects"[MeSH Terms])

**CINAHL search string**

(MH Critical Path or MH Practice Guidelines or PT (practice guidelines or standards or protocol or critical path or care plan) or TI ("position statement*" or "policy statement*" or "practice parameter*" or "best practice*") OR AB ("position statement*" or "policy statement*" or "practice parameter*" or "best practice*") or TI (standards or guideline or guidelines) or AB (practice N1 guideline* or treatment* N1 guideline*) or TI (CPG or CPGs) or TI consensus* or AB consensus* or AU (guideline* or standards or consensus* or recommendat*) or CA (guideline* or standards or consensus* or recommendat*) or TI (critical N2 path or critical N2 paths or critical N2 pathway or critical N2 pathways or critical N2 protocol* or clinical N2 path or clinical N2 paths or clinical N2 pathway or clinical N2 pathways or clinical N2 protocol* or practice N2 path or practice N2 paths or practice N2 pathway or practice N2 pathways or practice N2 protocol*) or AB (critical N2 path or critical N2 paths or critical N2 pathway or critical N2 pathways or critical N2 protocol* or clinical N2 path or clinical N2 paths or clinical N2 pathway or clinical N2 pathways or clinical N2 protocol* or practice N2 path or practice N2 paths or practice N2 pathway or practice N2 pathways or practice N2 protocol*) or TI recommendat* or TI (care N2 path or care N2 paths or care N2 pathway or care N2 pathways or care N2 map or care N2 maps or care N2 plan or care N2 plans or care N2 standard*) or AB (care N2 path or care N2 paths or care N2 pathway or care N2 pathways or care N2 map or care N2 maps or care N2 plan or care N2 plans or care N2 standard*) or TI (algorithm* AND (screening or examination or test or tested or testing or assessment* or diagnosis or diagnoses or diagnosed or diagnosing)) or AB (algorithm* AND (screening or examination or test or tested or testing or assessment* or diagnosis or diagnoses or diagnosed or diagnosing)) or (PT algorithm* AND TI (screening or examination or test or tested or testing or assessment* or diagnosis or diagnoses or diagnosed or diagnosing)) or ( PT algorithm* AND AB (screening or examination or test or tested or testing or assessment* or diagnosis or diagnoses or diagnosed or diagnosing)) or TI (algorithm* AND (pharmacotherap* or chemotherap* or chemotreatment* or therap* or treatment* or intervention*)) OR AB (algorithm* AND (pharmacotherap* or chemotherap* or chemotreatment* or therap* or treatment* or intervention*)) or (PT algorithm AND TI (pharmacotherap* or chemotherap* or chemotreatment* or therap* or treatment* or intervention*)) or (PT algorithm AND AB (pharmacotherap* or chemotherap* or chemotreatment* or therap* or treatment* or intervention*))) AND (XB ("position check") OR XB (verification) OR XB (validation) OR XB (confirmation) OR XB (determination) OR XB (control) OR XB (application) OR XB ("tube place determination") OR MH ("tube place determination") OR MH ("Catheter Placement Determination")) AND (XB ("OG tube") OR XB ("gastric lavage") OR XB ("enteral nutrition*") OR MH ("Nasoenteral Tubes") OR MH ("Intubation, Gastrointestinal") OR MH ("Feeding Tubes") OR MH ("Feeding Tube Care") OR MH (Gastric Lavage") OR XB ("nasogastric tube") OR XB ("nasogastric tube placement verification") OR XB (validation) OR XB ("enteral feeding tube") OR XB ("nasoenteral tube") OR XB ("gastric tube") OR XB ("stomach tube") OR XB ("enteral tube") OR XB ("feeding tube") OR XB ("gastrointestinal intubation") OR XB ("NG tube") OR XB ("orogastric tube“))

**Guideline registers screened**

| Agency for Healthcare Research and Quality (AHRQ) |
| --- |
| Arbeitsgemeinschaft der Wissenschaftlichen Medizinischen Fachgesellschaften e.V. (AWMF) |
| Belgian Health Care Knowledge Centre |
| British Association for Parenteral and Enteral Nutrition (BAPEN) |
| BIGG international database of GRADE guidelines |
| Canadian Medical Association Clinical Practice Guideline Infobase (CMA CPG Infobase) |
| Deutsches Netzwerk für Qualitätsentwicklung in der Pflege (DNQP) |
| Emergency Nurses Association Clinical Practice Guidelines |
| ERCI Guidelines Trust |
| Guideline Central |
| GIN (Guidelines International Network) |
| Haute Autorité de Santé (HAS) |
| Ministry of Health Singapore |
| NICE (National Institute for Health and Care Excellence) |
| NSW Health Policy Distribution System |
| Online Platform «Guidelines Schweiz» |
| Scottish Intercollegiate Guidelines Network (SIGN) |
| TRIP Database |

Table 4: Guideline registers screened

**References**

1. Brouwers M, Kho ME, Browman GP, Cluzeau F, Feder G, Fervers B et al. on behalf of the AGREE Next Steps Consortium. AGREE II: Advancing guideline development, reporting and evaluation in healthcare. Can Med Assoc J. Dec 2010, 182:E839-842; doi: 10.1503/cmaj.090449

2. Bankhead R, Boullata J, Brantley S, Corkins M, Guenter P, Krenitsky J et al.A.S.P.E.N. Enteral Nutrition Practice Recommendations. J Parenter Enter Nutr. 2009;33(2):122–67.

3. Boullata JI, Carrera AL, Harvey L, Escuro AA, Hudson L, Mays A et al. ASPEN Safe Practices for Enteral Nutrition Therapy. J Parenter Enter Nutr. 2017;41(1):15–103.

4. Koh P, Chin TS, Lee A, Tan P, Janet TP, Lai P et al. National guidelines on nursing management of nasogastric tube in adult patients. 2022.

5. Metheny NA. Initial and Ongoing Verification of Feeding Tube Placement in Adults (applies to blind insertions and placements with an electromagnetic device). Crit Care Nurse. 2016;36(2):e8–13.

6. NSW Health Government. Insertion and Management of Nasogastric and Orogastric Tubes in Adults. 2023;

7. Perry A, Kaiser J, Kruger K, Horigan AE, Bradford JY, Camarda A et al. ENA Clinical Practice Guideline Synopsis: Gastric Tube Placement Verification. J Emerg Nurs. 2024;50(2):301–4.
